# EAGP: an efficient generative augmentation framework for phage protein classification under severe class imbalance

**DOI:** 10.1093/bioinformatics/btag373

**Published:** 2026-06-15

**Authors:** Jiaru Li, Haoxiang Li, Yansu Wang, Quan Zou, Hongling Zhu

**Affiliations:** Faculty of Information Science and Engineering, Ocean University of China, Qingdao, Shandong 266100, China; Faculty of Information Science and Engineering, Ocean University of China, Qingdao, Shandong 266100, China; Institute of Fundamental and Frontier Sciences, University of Electronic Science and Technology of China, Chengdu, 610054, China; Institute of Digital Health, Yangtze Delta Region Institute (Quzhou), University of Electronic Science and Technology of China, Quzhou 324003, China; Institute of Fundamental and Frontier Sciences, University of Electronic Science and Technology of China, Chengdu, 610054, China; Institute of Digital Health, Yangtze Delta Region Institute (Quzhou), University of Electronic Science and Technology of China, Quzhou 324003, China; Shanghai Sixth People’s Hospital Affiliated to Shanghai Jiao Tong University School of Medicine, Shanghai, 200233, China

## Abstract

**Motivation:**

The accurate classification of phage proteins is critical for advancing bacteriophage research. Despite the proliferation of machine learning approaches in this domain, the persistent issue of data imbalance continues to hinder performance, particularly for rare protein sequences. Previous attempts to address this by re-weighting minority classes have faced limitations due to insufficient feature extraction capabilities.

**Results:**

In this paper, we introduce EAGP, a novel approach that integrates a generative model—functionally equivalent to a WGAN yet tailored for one-dimensional data—with the Evolutionary Scale Modeling (ESM) protein large language model for robust feature extraction. EAGP exhibits exceptional performance in binary classification and protein function annotation tasks. Crucially, our method not only improves overall classification efficacy but also significantly alleviates the performance degradation typically observed in minority classes.

**Availability and Implementation:**

The data and code underlying this article are available in GitHub at https://github.com/Innerly/EAGP and have been archived on Zenodo at https://doi.org/10.5281/zenodo.19928069.

## Introduction

Bacteriophages (phages), widely recognized as the most abundant biological entities in the biosphere, play a fundamental role in maintaining the ecological balance of microbial communities (Suttle 2005). Beyond their ecological significance, phages have garnered renewed attention as promising alternatives to antibiotics, particularly in the context of the escalating global crisis of antimicrobial resistance (AMR) (Kortright *et al.* 2019, Murray *et al.* 2022). Phage proteins, specifically virion proteins (PVPs) and receptor-binding proteins (RBPs), are critical determinants of the phage life cycle, facilitating host recognition and infection. Consequently, the accurate classification and functional annotation of these proteins are essential for elucidating phage-host interactions and advancing phage therapy applications (Leprince and Mahillon 2023).

Historically, the identification of phage proteins relied heavily on conventional experimental techniques, such as mass spectrometry and electron microscopy (Lavigne *et al.* 2009). While authoritative, these “wet-lab” methods are inherently labor-intensive, time-consuming, and expensive, making them ill-suited for the exponential growth of genomic data (Dion *et al.* 2020). To address these limitations, the field has shifted towards high-throughput computational approaches (Manavalan *et al.* 2019). In recent years, machine learning (ML) has emerged as a powerful paradigm for automated protein classification, offering a cost-effective and highly efficient alternative to traditional experiments (Bhaskar *et al.* 2006).

Existing computational methods for phage protein classification can be broadly categorized into two streams: traditional machine learning (Manavalan *et al.* 2017) and deep learning (DL) models. Traditional methods typically rely on manual feature engineering, where protein sequences are encoded into numerical vectors based on physicochemical properties or amino acid composition (e.g. AAC, DPC) (Chou 2009). Algorithms such as Random Forest (RF) (Kissanga *et al.* 2025), Support Vector Machines (SVM) (Manavalan *et al.* 2018b), and Logistic Regression have been widely deployed. While these models are computationally lightweight and interpretable, their performance is heavily constrained by the quality of handcrafted features, which often fail to capture the complex, high-dimensional patterns within protein sequences.

The advent of deep learning has revolutionized this domain by enabling automatic feature extraction. Neural network architectures, such as Convolutional Neural Networks (CNNs) and Recurrent Neural Networks (RNNs), have demonstrated superior capability in capturing latent sequence information (Almagro Armenteros *et al.* 2017, Min *et al.* 2017). Notable tools include DeePVP, which utilizes CNNs for sequence encoding (Fang *et al.* 2022), and recent transformer-based approaches like PhaVIP, which leverages Vision Transformers (ViT) for feature extraction (Shang *et al.* 2023). Furthermore, ProtPhage combines the ProtT5 protein language model with CNNs and Multi-Layer Perceptrons (MLP) to enhance classification accuracy (Elnaggar *et al.* 2021). Despite their high accuracy, these DL models are computationally intensive and require substantial resources for training and inference (Strubell *et al.* 2019).

Although current state-of-the-art (SOTA) methods have achieved remarkable progress, a persistent challenge remains: data imbalance. In phage proteomes, certain functional classes are significantly underrepresented compared to others (Yu *et al.* 2015). Standard data-driven models tend to bias towards the majority class, leading to poor generalization on minority (rare) proteins (Johnson and Khoshgoftaar 2019). Previous studies have attempted to mitigate this via cost-sensitive learning (re-weighting samples) (Krawczyk 2016). However, this approach merely forces the model to pay more attention to limited samples without introducing new information, often resulting in overfitting and limited feature diversity (Cao *et al.* 2019).

To overcome these bottlenecks, we propose EAGP, a novel framework that integrates the Evolutionary Scale Modeling (ESM) protein large language model with a generative data augmentation strategy specifically tailored for one-dimensional sequences. Unlike traditional oversampling methods, our approach employs a structure functionally equivalent to a Wasserstein Generative Adversarial Network (WGAN) to synthesize high-quality feature representations for minority classes (Adler and Lunz 2018). By leveraging the rich semantic knowledge embedded in ESM and the generative capability of WGAN, EAGP significantly enhances the robustness of classification. To clarify the meaning of “efficient” in this study, we note that EAGP adopts ESM-2 (650M) as the protein language model backbone, which has a smaller parameter scale than the ProtT5-based feature extractor used in ProtPhage. Therefore, the efficiency of EAGP mainly refers to achieving competitive or improved classification performance with a relatively smaller PLM backbone under the current experimental settings. The main contributions of this paper are summarized as follows:

1. We propose a unified framework combining pre-trained protein language models (ESM) with generative adversarial networks, achieving superior performance in overall phage protein classification tasks compared to existing baselines.
2. We address the critical issue of data imbalance by generating high-fidelity features for minority classes, significantly improving the model’s sensitivity and precision for rare proteins. It should be noted that the performance of EAGP is not solely attributable to the generative augmentation module. The ESM-2 encoder plays a fundamental role in extracting informative protein representations, while the generative augmentation module is designed to further alleviate class imbalance in the learned feature space.
3. We provide new insights into the application of generative data augmentation in bioinformatics, demonstrating that feature-space generation is a more effective strategy than simple sample re-weighting for protein sequence analysis.

## Related work

phANNs (Cantu *et al.* 2020) is a pioneering method that utilizes Logistic Regression for classification. Compared with other methods of its time, it has had a profound influence on subsequent phage classification research. Traditional machine learning methods significantly reduce the time cost of sequence analysis while maintaining high accuracy. With the rapid development of deep learning, there has been an increasing trend towards training deep models for phage protein classification.

DeePVP (Fang *et al.* 2022) introduced deep learning to this field by converting one-dimensional sequence data into two-dimensional image-like data. It employs Convolutional Neural Networks (CNNs) to extract features from these converted sequences. As results have shown, DeePVP achieved significant improvements in accuracy, precision, recall, and F1-score compared to traditional machine learning approaches.

While Transformers originated in Natural Language Processing (NLP), they have become the dominant architecture in deep learning. PhaVIP (Shang *et al.* 2023) successfully adapted the Vision Transformer (ViT) to extract features from phage protein sequences. Although it requires substantial computational resources, it delivers superior performance across metrics such as accuracy and F1-score. Given the rapid advancements in hardware capabilities, the focus has shifted towards prioritizing high classification capability despite the computational cost.

ProtPhage (Ou *et al.* 2025) represents a significant advancement in feature extraction, leveraging Pre-trained Protein Large Language Models (PLMs). Compared to basic deep learning methods, PLMs demonstrate strong generalization capabilities in feature extraction (Luo *et al.* 2025). ProtPhage pushed the performance of phage protein classification to a new peak. Furthermore, it employs a novel loss function to mitigate the data imbalance problem, achieving remarkable results.

In this work, we propose EAGP, a method that also leverages a pre-trained protein language model (ESM2) for feature extraction. However, to address the data imbalance problem, we employ a data augmentation strategy based on a generative augmentation module architecture designed for sequence data. Unlike ProtPhage, we choose to perform augmentation on the embeddings rather than the raw data. Since the embeddings extracted by ESM2 contain rich semantic features, performing augmentation in this latent space is more effective. This approach allows the model to generate new minority class samples that maintain high similarity to the original distribution, thereby enhancing the model’s ability to learn features from underrepresented classes.

## Methods

### Overview

The proposed EAGP framework integrates a pre-trained protein large language model (pLLM) with a Generative Adversarial Network (GAN)-based data augmentation module. The pre-trained pLLM functions as a feature encoder to extract high-dimensional representations from phage protein sequences. Subsequently, the data augmentation module is designed to mitigate the class imbalance problem. Crucially, we perform data augmentation on the embeddings generated by ESM-2 rather than on raw sequences. This strategy is adopted because the encoding process projects sequences into a latent space that captures richer semantic features and biological properties, thereby facilitating the generation of more realistic synthetic samples.

### Sequence encoding

In the first stage, EAGP processes the raw phage protein sequences by tokenizing them into individual amino acid residues. These tokens are then fed into the encoder. The primary objective is to extract discriminative features that capture the underlying biological syntax. Compared with traditional encoding methods, pre-trained protein language models offer superior precision and effectiveness. While sparse encoding schemes (e.g. One-Hot encoding) struggle to capture complex dependencies between amino acids, deep learning-based approaches can extract profound semantic information from protein sequences. Specifically, we employ ESM-2 (650M), which provides rich, context-aware protein representations learned from large-scale unlabeled sequences. It captures both local biochemical patterns and long-range evolutionary dependencies without requiring multiple sequence alignments. This property makes ESM-2 (650M) particularly suitable for phage proteins, which often lack reliable homologs and annotated domains. For sequence preprocessing, all protein sequences were converted to uppercase and whitespace was removed. Ambiguous or rare amino acid symbols, including X, B, Z, J, U, and O, were retained as valid tokens, while other unsupported characters were replaced with X. The cleaned sequences were tokenized by the ESM-2 (650M) tokenizer with a maximum length of 1022 tokens. Longer sequences were truncated from the C-terminal side, thereby retaining the N-terminal part, whereas shorter sequences were padded when necessary according to the tokenizer’s default padding strategy. Protein sequences were first cleaned to remove any non-standard amino acid characters. Sequences exceeding the maximum input length supported by the ESM-2 language model (650M) were truncated to fit the model. Specifically, for sequences longer than the model’s maximum token limit, only the first *N* amino acid residues from the N-terminal were retained, as this region typically contains critical structural signals for virion proteins. Alternatively, for analyses requiring full coverage, a sliding-window or chunking strategy could be applied, where overlapping segments of the sequence are encoded separately and subsequently aggregated. For sequences shorter than the model’s required input length, padding was applied to maintain consistent input dimensions. Padding tokens were appended to the C-terminal of the sequence to reach the fixed length, ensuring proper alignment in batch processing without introducing artificial signals. The model was trained and evaluated using these processed sequences, and all embeddings were generated consistently according to this preprocessing protocol. This approach ensures reproducibility of the deep learning pipeline and proper handling of sequence length variability in phage proteomes.

### Data augmentation

**Figure 1:**
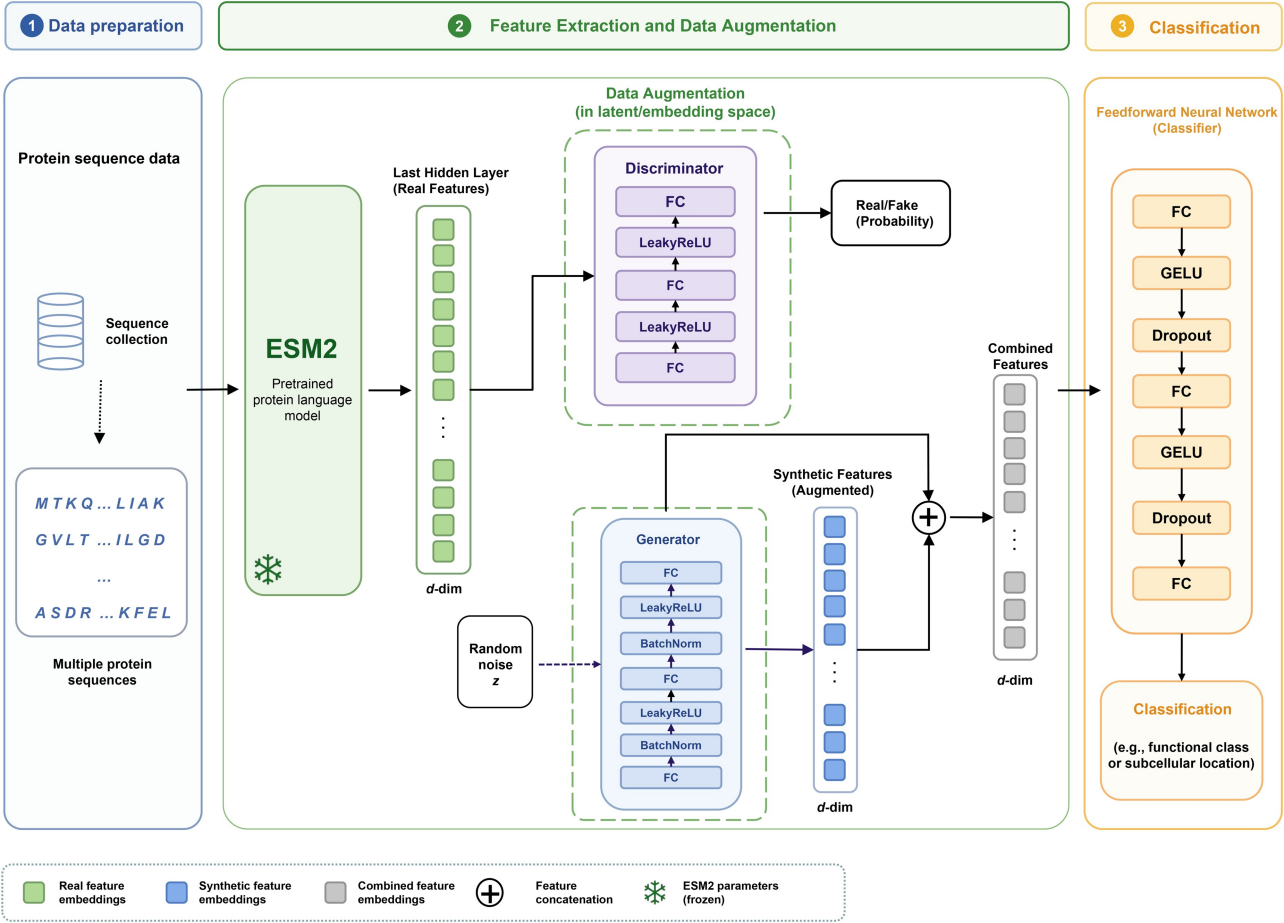
The overall architecture of the proposed deep learning framework integrating ESM2 feature extraction and generative data augmentation.

As illustrated in Fig. 1, our data augmentation module consists of two primary components: a Generator and a Discriminator. To address the data imbalance, we first analyze the distribution of the seven protein classes and identify the top-*k* minority classes with the fewest samples. The embeddings of these minority classes serve as the training data for the Generator, which learns to synthesize new, realistic embedding samples. The Discriminator is responsible for distinguishing between the generated embeddings and the real embeddings from the original minority classes. Through adversarial training, the module enriches the feature space of minority classes, thereby improving the classifier’s robustness. The embeddings generated by generative augmentation module are used to augment the original training set rather than replace it. Before classifier training, the generated minority-class embeddings are mixed with the original real embeddings, and the classifier is then trained on the augmented dataset.

In practice, the generator is updated once every $n_{G}$ discriminator updates, with $n_{G}=5$ in our implementation. Both the generator and the discriminator are optimized using the Adam optimizer with a learning rate of $1\times 10^{-4}$ and momentum parameters $\left(\beta_{1},\beta_{2})=(0.5,0.9\right)$. Training is performed for a fixed number of epochs, and all gradients are computed via automatic differentiation.

To address data imbalance, we first analyzed the distribution of the seven structural protein classes and selected the top-k minority classes with the fewest training samples. In our experiments, k was set to 2, meaning that the two least-represented classes were used for generative augmentation module.

## Results

### Overview

To provide a more interpretable evaluation of EAGP, we followed three commonly used experimental settings that capture key sources of difficulty in phage protein annotation. The temporal split evaluates temporal generalization by testing whether models trained on earlier data remain effective on proteins collected from later time periods. The sequence-similarity-controlled setting examines model robustness under increasing sequence divergence, which is particularly relevant for annotating proteins with limited homology to known examples. The imbalance-ratio setting assesses model performance as class distributions become progressively skewed, reflecting the under-representation of certain structural protein categories in real-world phage datasets. By framing the results under these established settings, we aim to connect the observed performance differences with specific biological and computational challenges, rather than presenting them solely as numerical comparisons. The ProtPhage results were taken from the original ProtPhage publication. Therefore, although these results provide a useful reference for comparison with a representative state-of-the-art method, they may not fully reflect differences under an identical hardware, software, and retraining environment.

### Performance on the benchmark dataset split by time

The time-based split was designed to evaluate the temporal generalization ability of EAGP. In practical annotation scenarios, newly deposited phage proteins may differ from proteins available during model development. Therefore, this setting provides a more realistic evaluation of whether the model can maintain reliable performance on future or recently collected sequences rather than relying only on randomly mixed training and test samples. To assess the model’s generalization capability under realistic deployment scenarios, we adopted a temporal data splitting strategy. In this setup, sequences identified earlier were utilized for training, while subsequently discovered sequences were reserved for testing. This approach simulates the prospective application of the model to novel phage sequences.

Table 1 presents the performance on the binary classification task (PVP vs. non-PVP) under this temporal split. EAGP outperforms all comparison methods, achieving an accuracy and F1-score of 0.9796 and 0.9797. Notably, EAGP surpasses the ProtPhage baseline (0.9717 and 0.9673) even without the use of data augmentation in this balanced binary setting. This performance gain is primarily attributed to the superior feature extraction capabilities of the ESM-2 protein language model. Unlike the ProtT5 model used in ProtPhage, ESM-2 generates embeddings that capture richer evolutionary semantics, enabling the classifier to effectively distinguish PVPs from non-PVPs solely based on learned representations. Notably, EAGP surpasses the ProtPhage baseline even without the use of data augmentation in this balanced binary setting. This performance gain is primarily attributed to the superior feature extraction capabilities of the ESM-2 protein language model.

**Table 1:** Performance on the benchmark dataset split by time (binary PVP vs. non-PVP classification).

Method	Acc	Prec	Rec	F1
phANNs	0.9050	0.8917	0.8845	0.8881
PhaVIP	0.9250	0.8945	0.9343	0.9140
ProtPhage	0.9717	0.9528	**0.9823**	0.9673
EAGP (ours)	**0.9796**	**0.9797**	0.9796	**0.9797**

Best results in each group are marked in bold.

**Figure 2:**
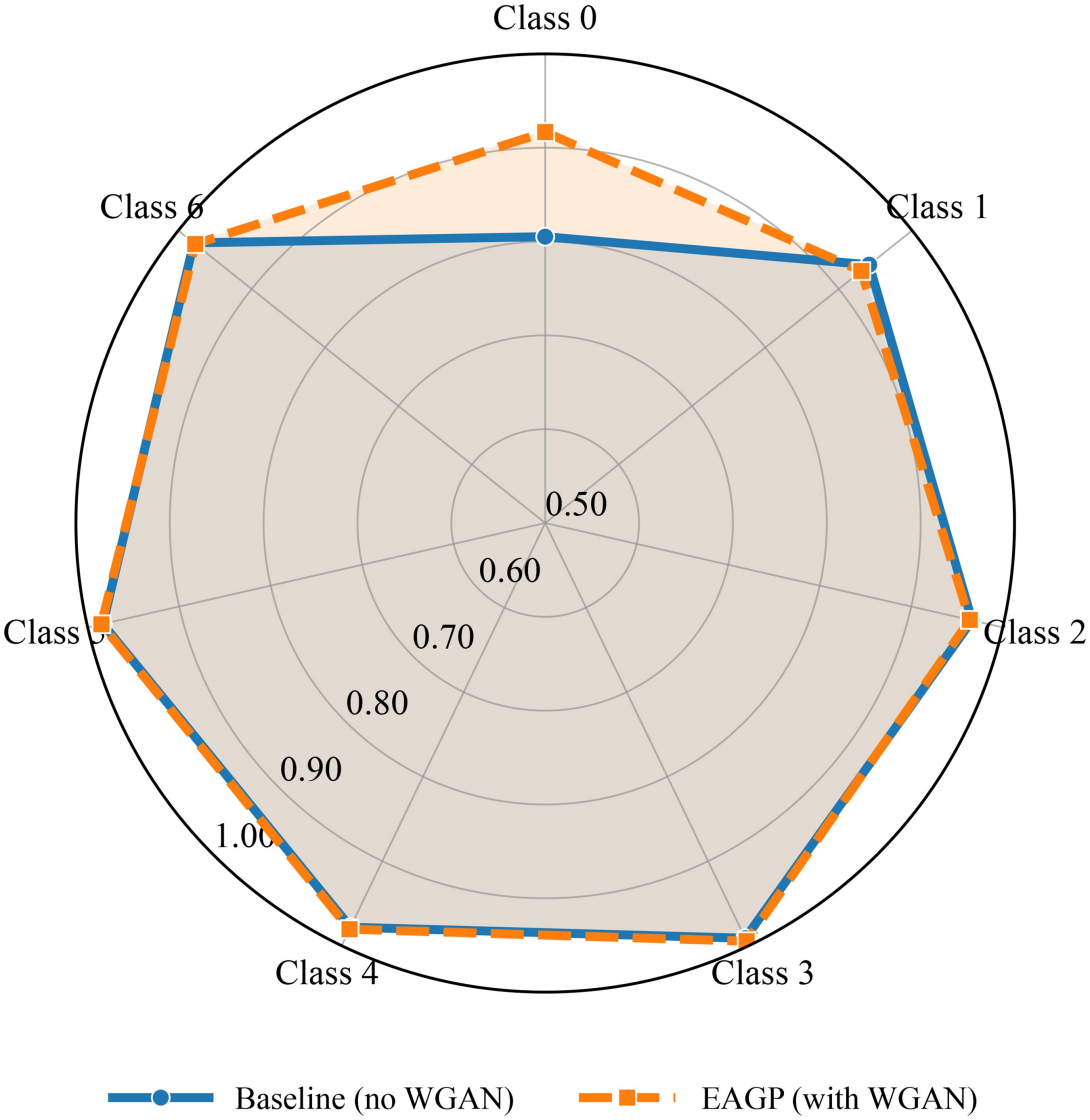
F1 scores for the seven structural protein classes predicted by EAGP. Class 0–Class 6 correspond to the following protein types: Class 0, minor capsid; Class 1, tail fiber; Class 2, major tail; Class 3, portal; Class 4, minor tail; Class 5, baseplate; Class 6, major capsid.

**Figure 3:**
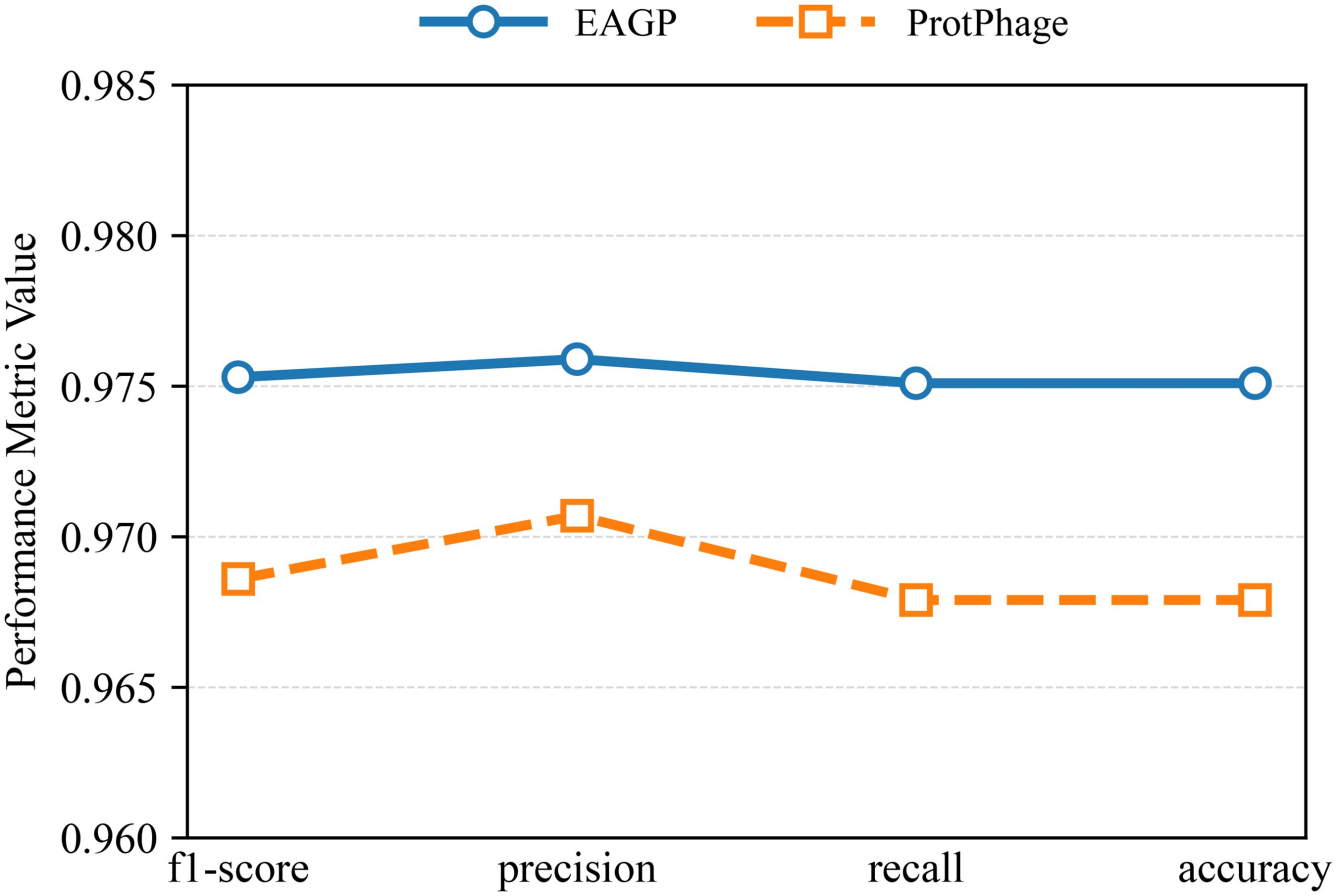
Performance comparison between the proposed EAGP model and the ProtPhage method across four evaluation metrics.

The benchmark dataset used in this study contains 18 018 protein sequences, including 9582 sequences for training, 4107 sequences for validation, and 4329 sequences for testing; the detailed sample-size distribution across the seven structural protein classes is provided in the released code repository. Furthermore, we evaluated the efficacy of the generative augmentation module generative augmentation module in mitigating class imbalance, a critical challenge in multi-class scenarios. To isolate the contribution of the augmentation strategy, we conducted an ablation study comparing EAGP (ESM-2 + WGAN) against a baseline ESM-2 model without augmentation. As illustrated in Fig. 2, the unaugmented baseline struggles with minority classes, yielding an F1-score of only 0.80 for Class 0. In contrast, integrating generative augmentation module significantly elevates the F1-score for Class 0 to 0.91. This improvement of 0.11 suggests that the synthetic embeddings generated by the WGAN-GP module may help enrich the minority-class feature space and reduce the classifier’s bias toward majority classes under this experimental setting, alleviating bias toward majority classes. Crucially, as shown in Fig. 3, this enhancement in minority class performance does not compromise the accuracy of majority classes, which maintain robust F1-scores exceeding 0.975. Collectively, these findings demonstrate that EAGP exhibits strong robustness against both temporal distribution shifts and class imbalance. The consistently strong performance under the time-based split indicates that EAGP retains robust predictive ability on temporally separated phage proteins. This suggests that the framework is less dependent on dataset-specific random partitioning and may be better suited for real-world phage protein annotation tasks where newly discovered sequences are continuously added to public databases.

### Performance on the benchmark dataset split by similarity

The sequence similarity analysis was conducted to examine whether EAGP mainly benefits from highly similar homologous proteins or whether it can still identify PVPs and structural categories when sequence similarity decreases. This is particularly important for phage protein annotation because many newly discovered phage proteins show low similarity to known sequences. We further assessed the model’s generalization ability across varying sequence identity thresholds. As shown in Table 2, EAGP exhibits excellent binary classification performance for identifying PVPs. Even at a stringent sequence similarity threshold of 40, EAGP maintains an F1-score of 0.9821, demonstrating high accuracy in distinguishing PVPs from non-PVPs. As the similarity threshold increases, the model’s performance remains stable, with F1-scores fluctuating slightly between 0.9750 and 0.9872, indicating strong robustness against sequence homology variations.

**Table 2:** Binary PVP identification performance across sequence-identity thresholds.

Threshold	Method	ACC	Precision	Recall	F1
40	ProtPhage	0.9813	**0.9846**	0.9778	0.9812
	EAGP (ours)	**0.9821**	0.9821	**0.9821**	**0.9821**
50	ProtPhage	0.9690	**0.9865**	0.9510	0.9684
	EAGP (ours)	**0.9720**	0.9725	**0.9720**	**0.9720**
60	ProtPhage	0.9741	**0.9858**	0.9621	0.9738
	EAGP (ours)	**0.9750**	0.9753	**0.9750**	**0.9750**
70	ProtPhage	0.9812	**0.9826**	0.9799	0.9812
	EAGP (ours)	**0.9825**	0.9825	**0.9825**	**0.9825**
80	ProtPhage	0.9725	**0.9868**	0.9579	0.9721
	EAGP (ours)	**0.9764**	0.9767	**0.9764**	**0.9764**
90	ProtPhage	0.9820	**0.9886**	0.9752	0.9819
	EAGP (ours)	**0.9872**	0.9873	**0.9872**	**0.9872**

Best results in each group are marked in bold.

It is worth noting that for the binary classification task, the number of PVP and non-PVP samples is relatively balanced. Therefore, we opted not to employ generative augmentation module for data augmentation in this specific sub-task to optimize computational efficiency. This flexible design allows for rational resource allocation, ensuring that computationally intensive data augmentation is reserved for tasks with severe class imbalance, specifically the multi-class PVP function annotation.

**Figure 4:**
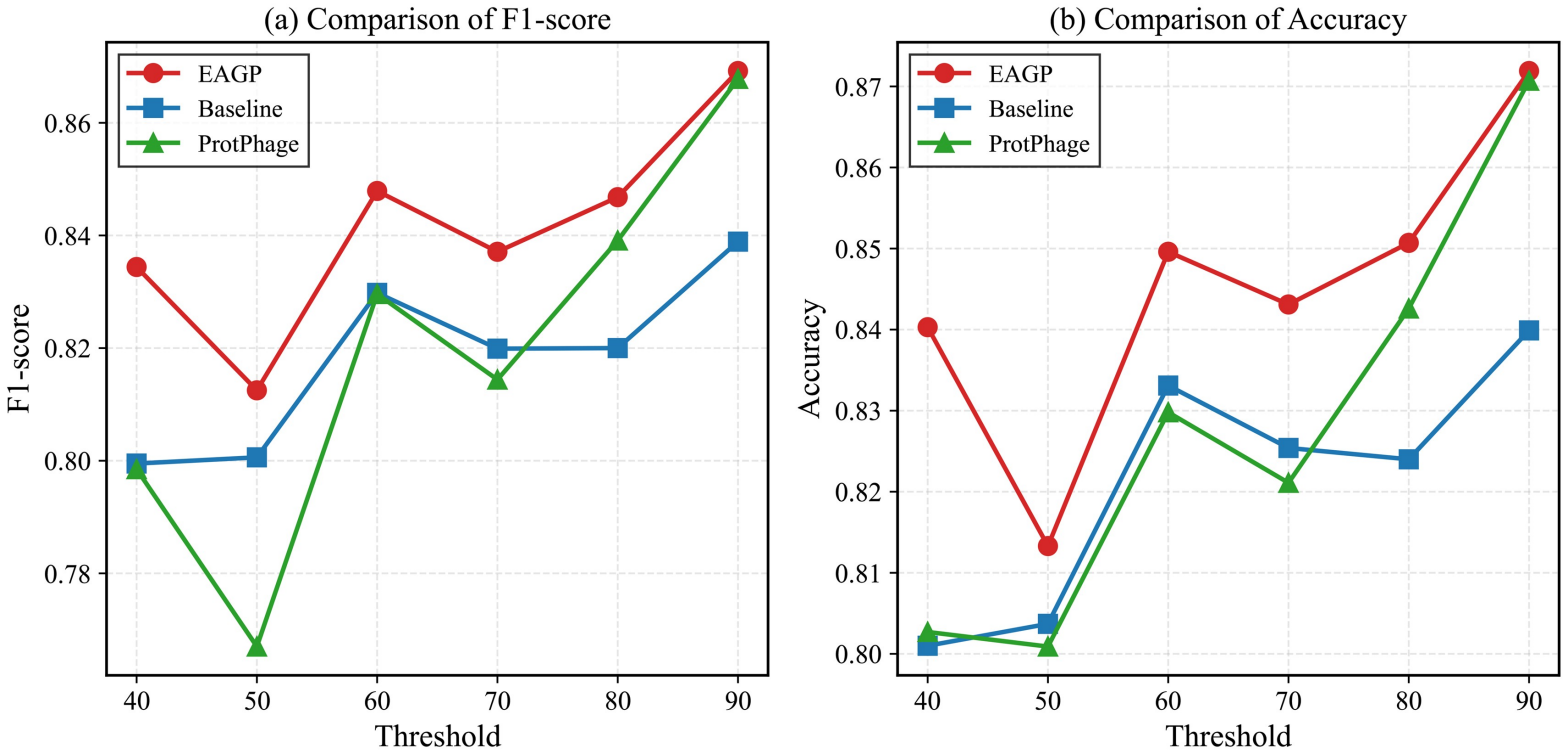
Performance comparison of the proposed EAGP model against Baseline and ProtPhage under different threshold settings. (a) Comparison of F1-score across thresholds ranging from 40 to 90. (b) Comparison of Accuracy across the same threshold range.

Figure 4 presents model performance across varying sequence identity thresholds (ranging from 40 to 90) to evaluate generalization capability on effectively novel sequences. EAGP exhibits relatively stable performance in low-homology scenarios; specifically, in the challenging 40 and 50 identity intervals, the cost-sensitive ProtPhage suffers a significant decline with its F1-score dropping below 0.77, whereas EAGP maintains a robust F1-score exceeding 0.81. This contrast suggests that while re-weighting strategies may overfit to specific minority samples, our generative augmentation strategy synthesizes diverse features that enable better generalization to distant evolutionary relatives. Furthermore, the contribution of the generative module is evident across the entire spectrum, as EAGP consistently surpasses the non-augmented Baseline model (ESM-2). For instance, the clear performance gain at the 60 threshold confirms that generative augmentation module synthesized embeddings may provide additional minority-class feature coverage and improve the decision boundary beyond the capabilities of the pre-trained model alone. Finally, unlike ProtPhage, which demonstrates high volatility (e.g. sharp fluctuations between 50 and 60), EAGP maintains a smoother, consistently high-performance trajectory, offering the stability critical for practical phage annotation where sequence similarity to known databases varies unpredictably. The results across different sequence similarity thresholds show that EAGP maintains stable performance even when sequence similarity is reduced. This finding suggests that the ESM-2-based representations, combined with the generative augmentation strategy, enable EAGP to capture functional signals beyond simple sequence-level similarity. Therefore, the model is potentially useful for annotating divergent or less-characterized phage proteins.

### Performance on the benchmark dataset split by imbalance

**Figure 5:**
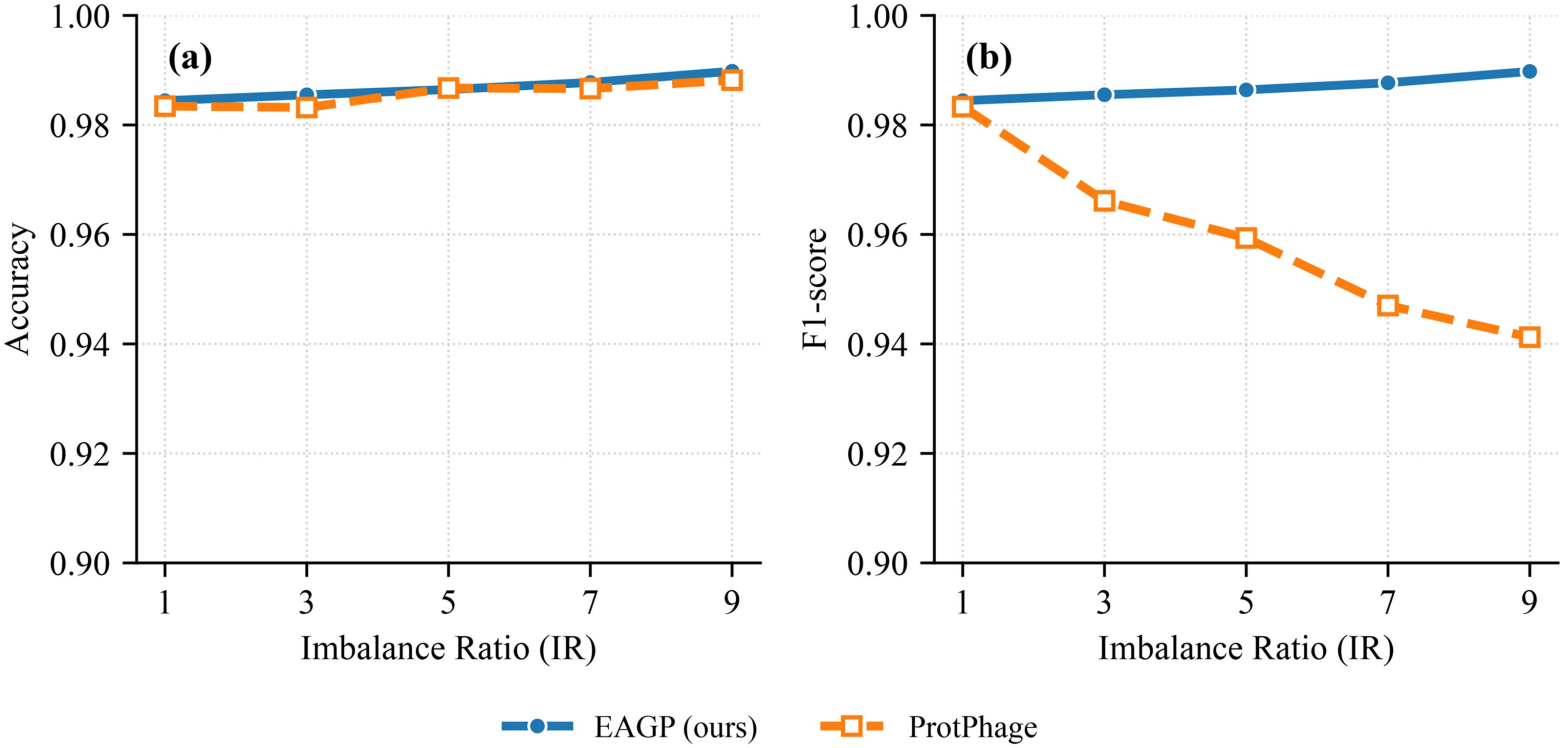
Performance stability analysis of EAGP and ProtPhage under varying imbalance ratios. (a) Comparison of Accuracy as the Imbalance Ratio (IR) increases from 1 to 9. (b) Comparison of F1-score under the same IR conditions. Note: The blue solid line represents the proposed EAGP model, while the orange dashed line denotes the ProtPhage method. The x-axis represents the ratio of negative to positive samples (Imbalance Ratio).

The imbalance-ratio experiment was designed to evaluate the robustness of EAGP under different degrees of class imbalance. This setting is directly relevant to phage protein functional annotation, where some structural categories, such as tail fiber or baseplate proteins, are often underrepresented compared with more abundant classes. To rigorously test the model’s resilience to data disparity, we evaluated performance across increasing Imbalance Ratios (IR) ranging from 1 to 9. As shown in Fig. 5a, both models achieve high accuracy, consistently remaining above 0.98 regardless of the imbalance level. However, accuracy can be a misleading metric in imbalanced scenarios.

Figure 5b reveals the distinct advantage of the proposed method regarding the F1-score. While the baseline model (ProtPhage) shows a marked performance degradation—dropping from an F1-score of approximately 0.98 at IR = 1 to roughly 0.94 at IR = 9. EAGP demonstrates exceptional stability. Our method maintains an F1-score consistently exceeding 0.98 across all tested ratios. These results confirm that the ESM2 embeddings combined with our augmentation strategy effectively prevent the model from biasing toward the majority class, ensuring robustness even under severe class disparity. The improved performance of EAGP under imbalanced settings demonstrates that the proposed generative augmentation module can alleviate the negative impact of minority-class scarcity. In particular, the gains observed for underrepresented structural categories suggest that WGAN-based feature augmentation enriches the representation space of minority classes and enables the classifier to better distinguish rare but biologically important PVP subclasses.

### Case study: annotating proteins on the mycobacteriophage PDRPxv genome

EAGP is designed to address the challenge of phage protein classification, facilitating the functional annotation of bacteriophage genomes. To validate the practical utility and robustness of our model, we conducted a case study using the *Mycobacterium* phage PDRPxv genome Sinha *et al.* (2020) previously characterized this genome and identified 107 protein sequences. Using this experimentally validated dataset as a benchmark is critical for assessing the meaningfulness and real-world applicability of our research.

We first evaluated the performance of EAGP on the Phage Virion Protein (PVP) identification task. As shown in Table 3, EAGP demonstrated superior classification capability, successfully identifying all 12 confirmed PVPs (12/12). This performance surpasses phANNs, DeePVP, and PhaVIP, and matches the accuracy of ProtPhage.

**Table 3:** Comparison of PVP identification methods on the PDRPxv genome.

Method	PVP Identification Rate
phANNs	7/12
DeePVP	9/12
PhaVIP	11/12
ProtPhage	12/12
EAGP	12/12

Furthermore, Table 4 details the specific functional predictions made by EAGP compared to the putative functions reported by Sinha *et al.* (2020). The results indicate that EAGP can accurately predict the specific roles of structural proteins. This demonstrates that EAGP possesses robust generalization capabilities and holds significant potential for downstream applications in phage genomic analysis.

**Table 4:** Comparison of EAGP and ProtPhage predictions with putative functions for PDRPxv proteins.

Protein ID	EAGP Prediction	ProtPhage Prediction	**Putative Function (** Sinha *et al.* 2020 **)**
Gp8	Portal	Portal	Portal
Gp10	Portal	Portal	Minor head
Gp11	Tail fiber	Baseplate	Scaffolding
Gp12	Major capsid	Major capsid	Major capsid
Gp18	Major tail	Major tail	Major tail
Gp25	Minor tail	Minor tail	Tail assembly chaperone
Gp28	Minor tail	Tail fiber	Tape measure
Gp29	Minor tail	Minor tail	Minor tail
Gp30	Minor tail	Minor tail	Minor tail
Gp31	Minor tail	Minor tail	Minor tail
Gp32	Minor tail	Minor tail	Minor tail
Gp33	Minor tail	Minor tail	Minor tail

Overall, EAGP showed relatively high consistency with the reported putative annotations for several structural protein categories, particularly minor tail proteins and major structural components such as portal, major capsid, and major tail proteins. These observations suggest that EAGP can provide useful genome-level structural protein annotations and may support subsequent downstream analyses of phage proteomes.

### Using EAGP-classified structural proteins to support phage host prediction

Based on the critical role of bacteriophages in combating bacterial infections, research on phage host prediction is highly significant for advancing human health. Accurate host prediction relies on precise identification and classification of structural proteins within phage proteomes.

To evaluate this, we applied our model, EAGP, to this task and observed that it achieves very high accuracy, as summarized in Table 5.

**Table 5:** EAGP-based identification of PVPs and tail fiber proteins in representative phage proteomes.

Phage	Predicted PVPs/Total proteins	Predicted tail fiber proteins	Correct?
*Escherichia* phage T4	50/278	11	✓
*Klebsiella* phage KP32	19/44	7	✓
*Salmonella* phage P22	13/72	4	✓
*Staphylococcus* phage phi 11	17/53	3	✓

PVP, phage virion protein. “Correct” indicates whether EAGP successfully identified at least one tail fiber protein consistent with the curated structural annotation of the corresponding phage proteome.

We selected representative phages of medical and biological relevance, including Escherichia phage T4, Klebsiella phage KP32, Salmonella phage P22, and Staphylococcus phage phi 11. After data preparation, protein sequences were passed through the ESM2 encoding module to generate embeddings for each protein. These embeddings were then input into our pre-trained classifiers. In the first stage, EAGP performs a binary classification to distinguish PVPs from non-PVPs. Proteins predicted as non-PVP are filtered out, while predicted PVPs are forwarded to a second classifier for functional annotation.

Tail fiber proteins are known to play a crucial role in host recognition (Gonzalez-Serrano *et al.* 2023), making their accurate annotation essential for downstream host prediction. In this study, we specifically tracked the number of tail fiber proteins correctly annotated by EAGP.

As shown in Table 5, EAGP demonstrates outstanding performance in both binary PVP classification and multi-class functional annotation. Proper functional annotation of structural proteins is vital for downstream models, such as the RBP-based host prediction method described by Boeckaerts *et al.* (2021), to accurately infer phage hosts. These results highlight the considerable potential of EAGP to support phage-host prediction pipelines by providing reliable structural protein annotations.

## Conclusion

In this work, we introduce EAGP, a unified framework that combines pre-trained protein language models with WGAN-GP-based generative augmentation for phage protein classification under class imbalance. EAGP addresses minority-class scarcity by generating additional feature representations in the ESM-2 embedding space. Across benchmark datasets stratified by time, sequence similarity, and class imbalance, EAGP achieves improved or competitive performance compared with existing methods under the current experimental settings. In the PDRPxv case study, EAGP correctly identified all confirmed PVPs and provided structural protein annotations that were partially consistent with reported putative functions. These results suggest that feature-space generative augmentation may be a useful strategy for improving phage protein classification in imbalanced scenarios. Nevertheless, further validation on larger and more diverse phage proteomes will be needed to assess the generalizability and biological reliability of the generated feature representations.

Although EAGP demonstrates strong performance in phage protein sequence classification, it still has several limitations. First, EAGP requires relatively higher computational resources than conventional machine learning models, such as Random Forest (Manavalan *et al.* 2018a). Second, similar to many deep learning-based approaches, the interpretability of EAGP remains limited, making it difficult to clearly explain every step involved in the model’s decision-making process.

## Data Availability

The data and code underlying this article are available in GitHub at https://github.com/Innerly/EAGP and have been archived on Zenodo at https://doi.org/10.5281/zenodo.19928069.
